# Notes and News

**Published:** 1905-11-18

**Authors:** 


### NOTES AND NEWS.

THE Hunterian Museum has been redecorated at a cost of £1,100.

PRINCESS CHRISTIAN will open the new buildings at the Hampstead Hospital on December 16.

THE Harvey Laboratory at King's School, Canterbury, will be opened on November 18. Sir Douglas Powell will attend and speak on the occasion.

THE late Mr. Charles Pocock, of Camberwell, has bequeathed the residue of his estate, amounting to between £5,000 and £6,000, to three London hospitals.

A CONCERT in aid of the Paddington Green Hospital for Children will be given on November 28 at the Kensington Town Hall. Mr. Lewis Waller, Miss Mary Moore, and other well-known artistes will give their services.

THROUGH the will of Mr. Charles Ansell, of Sloane Street, many charities are provided for should his son die without issue. The provisional bequests include £50,000 to the King's Fund, and £10,000 each to a number of London hospitals.

DR. CHILDS read an interesting paper on "The Lincoln, Maidstone, and Worthing Typhoid Epidemics" before the Incorporated Society of Medical Officers of Health last week. A discussion followed, from which it was evident that the recognition of the necessity for more supervision of water supplies was general.

REGULATIONS have been made for the Begby Scholarship in connection with the Royal College of Surgeons, founded by Mrs. Begby, in memory of her husband, a former member of the College. The Scholarship, of the value of £20 per annum, will be open to any candidate admitted to the second examination of the Joint Examining Board of England held in 1906. It will be awarded to the student obtaining the highest marks in anatomy.

MR. HENRY T. BUTLIN will deliver the Bradshaw Lecture on December 13. The subject will be "Carcinoma is a Parasite Disease."

THROUGH the will of the late Col. Maurice Smith, of Cambridge Terrace, the King's Fund will receive £200 now, and £1,000 at a later date.

A NEW pavilion has been opened at the Bradford Union Hospital. It will provide accommodation for 153 patients.

THE Chaplain of the Tottenham Hospital has sent us a "Hymn for the King." The verses are evidently inspired by a loyal and devout spirit, and are set to music by G. Drewett. The Chaplain offers to send the hymn to anyone who writes for it.

AT the meeting of the Royal Statistical Society on Tuesday, Mr. R. Rew was presented with the Guy Medal for his work in connection with the Special Committee to investigate the Production and Consumption of Meat and Milk in the United Kingdom and for his paper on "Observations on Production of meat and Dairy Products."

A SPECIAL general meeting of the Royal Medical and Chirurgical Society will be held at 20 Hanover Square on Thursday, November 23, at five o'clock. The meeting will consider a report on the Union of Medical Societies as amended and adopted at the meeting of the General Committee of the Representatives of the Societies in July 1905.

A MEETING of the Council of the Invalid Children's Aid Association will be held on Thursday, November 30, at 4 P.M., at Denison House, Vauxhall Bridge Road, when Sir William Broadbent will give an address on "The Tuberculosus Children of the Metropolis," and a report will be given of the work of the Association and its possible development.

A GRAND American bazaar will be held in aid of the West Ham Hospital in the Stratford Town Hall on November 23 to 25. The Duke and Duchess of Connaught will open the bazaar on the first day, the American Ambassador on the second day, and the Japanese Ambassador on the third day. The Duchess of Marlborough, President of the Hospital, will hold a stall.

THE honours conferred on the occasion of the King's birthday include the following: Dr. James Barr, Physician to the Royal Infirmary, Liverpool, and Mr. Arthur Chance, President of the Royal College of Surgeons, Ireland, received knighthood. Dr. Theodore Thomson, a medical officer of the Local Government Board, Dr. Marc Armand Ruffer, President of the Egyptian Sanitary Board, and Dr. Featherston Cargill, Resident in the Protectorate of Northern Nigeria, were made C.M.G. Sir Felix Semon, Physician Extraordinary to the King, has been advanced to the knighthood of the Victorian Order. Mr. John McFadyean, of the Royal Veterinary College, has been knighted.

